# Effects of Phosphogypsum–Recycled Aggregate Solid Waste Base on Properties of Vegetation Concrete

**DOI:** 10.3390/ma19010014

**Published:** 2025-12-19

**Authors:** Zhan Xiao, Nianchun Deng, Mingxuan Shen, Tianlong Wang, Xiaobing Chen, Shuangcan Li

**Affiliations:** 1 School of Civil Engineering and Architecture, Guangxi University, Nanning 530004, China; 17885675703@163.com; 2 Guiyang Urban Construction Engineering Group Co., Ltd., Guiyang 550002, China; 3 School of Civil Engineering, Guizhou University, Guiyang 550025, China; zienshen@126.com; 4 School of Management, Guizhou University, Guiyang 550025, China; tl_wang03@163.com

**Keywords:** phosphogypsum, recycled aggregate, vegetation concrete, solid waste recycling, plant compatibility, strength, alkalinity, microscopic analysis

## Abstract

Vegetation concrete is a composite material integrating plant growth and concrete technology. In this study, solid waste materials (phosphogypsum and recycled aggregates) were utilized to prepare vegetation concrete. Semi-hydrated phosphogypsum (HPG) was used to replace ordinary Portland cement as a cementitious material in a gradient manner, while recycled coarse aggregates (RCAs) fully replaced natural crushed stone. The basic properties of phosphogypsum–recycled aggregate-based vegetation concrete were analyzed, and X-ray diffraction (XRD) and scanning electron microscopy (SEM) were employed to characterize the hydration products of vegetation concrete with different mix ratios. The results indicated that replacing cement with HPG exerted a significant alkali-reducing effect and provided favorable cementitious strength. When the porosity was 24% and the HPG content was 50%, the vegetation concrete exhibited optimal performance: the 28-day compressive strength reached 12.3 MPa, and the pH value was 9.7. Recycled aggregates had a minimal impact on strength. When 0.5% sodium gluconate was added as a retarder, the initial setting time was 97 min and the final setting time was 192 min, which met construction requirements with little influence on later-stage strength. Microscopic analysis revealed that the early strength (3d–7d) of vegetation concrete was primarily contributed by CaSO_4_·2H_2_O crystals (the hydration product of HPG), while the later-stage strength was supplemented by C-S-H (the hydration product of cement). Planting tests showed that Tall Fescue formed a lawn within 30 days; at 60 days, the plant height was 18 cm and the root length was 6–8 cm. Some roots grew along the sidewalls of concrete pores and penetrated the 5 cm thick vegetation concrete slab, demonstrating good growth status.

## 1. Introduction

Vegetation Concrete is a composite material that combines plant growth and concrete technology, featuring multiple functions such as structural support, plant greening, soil and water loss prevention, water filtration and purification, and light weight [1,2,3]. It is mainly composed of coarse aggregates, cementitious materials, admixtures, and water [4], forming a porous concrete structure dominated by connected pores [5]. The roots of the vegetation layer penetrate into the pores and extend to the soil at the bottom of the concrete, realizing the organic integration of plant root growth, soil and water conservation, and ecological restoration. It has been widely applied in slope protection, riverbank revetment, parking lots, roof greening, and other scenarios [6,7,8].

The core properties of vegetation concrete include the following:

- Mechanical strength must meet engineering structural requirements [9];
- Pore structure (porosity, pore size distribution) must ensure water penetration, root growth, and air circulation [10];
- Alkalinity (pH value) must adapt to the low-alkalinity environment required for plant growth [11,12].

Among these, the balance between mechanical strength and porosity, as well as the control of pH value, are current research focuses and challenges for vegetation concrete.

To ensure strength, conventional vegetation concrete mostly uses cement as the cementitious material. However, the hydration of the cement matrix produces a large amount of OH^−^, and its high alkalinity inhibits plant growth [13], and may also cause water pollution, soil alkalization, and other problems in the ecosystem. Therefore, alkali reduction treatment is necessary. Cao et al. [14] adopted low-alkali slag sulfoaluminate cement as a replacement for ordinary Portland cement (OPC), which significantly reduced the alkalinity of vegetation concrete, resulting in a pH value below 8.5 at 28 days. Zhao et al. [15] utilized magnesium phosphate cement (MPC) instead of OPC to prepare porous ecological concrete, and the MPC-based ecological concrete exhibited superior vegetation performance compared with the OPC-based counterpart. Nevertheless, the high cost of low-alkali cement hinders its popularization [16]. Ganapathy et al. [17] demonstrated that the addition of silica fume (SF) and fly ash (FA) can consume calcium hydroxide (CH), thereby reducing the pH value of vegetation concrete. Xie et al. [18] adopted the soaking method with solutions such as NH_4_HCO_3_, KH_2_PO_4_, and NH_4_H_2_PO_4_ for alkalinity reduction. The results indicated that all three solutions could rapidly decrease the pore solution pH to below 7 within 1 day and maintain it in the range of 7.0–8.5 over 10 days. Wang et al. [19] demonstrated that spraying silane on vegetation concrete achieved a significant alkalinity reduction in the early stage; however, as the curing age increased, Ca(OH)_2_ was gradually generated and diffused inside the concrete, leading to a gradual rise in alkalinity. Liu et al. [20] prepared a soil amendment by blending fly ash, vinyl acetate emulsion, etc., and mixed it with humus soil and vermiculite powder at a certain ratio to form a soil matrix. Tests showed that despite the pore environment of the vegetation concrete having a pH of 11–12, Tall fescue exhibited satisfactory growth performance. These methods have a certain alkali-reducing effect but may damage the mechanical strength of vegetation concrete, and their complex processes are not conducive to construction.

In this study, solid waste phosphogypsum and recycled aggregates were used to replace cement and natural crushed stone, respectively, for preparing vegetation concrete. Phosphogypsum (PG) is an industrial by-product of wet-process phosphoric acid production. The global annual discharge of PG is 190–290 million tons [21], with a comprehensive utilization rate of only approximately 25% [22]. The extensive stacking of PG causes environmental pollution and resource waste. As phosphoric acid tailings, PG mainly consists of dihydrate calcium sulfate (CaSO_4_·2H_2_O). Due to the production process, it contains impurities such as residual soluble phosphorus (P_2_O_5_), soluble fluorine (HF), free phosphoric acid (H_3_PO_4_), and sulfuric acid (H_2_SO_4_) [23], with a pH value of 2–3 (acidic), which easily causes environmental pollution [24,25]. However, dihydrate phosphogypsum can be converted into semi-hydrated phosphogypsum (CaSO_4_·0.5H_2_O) through calcination or drying-dehydration [26]. It possesses cementitious properties as it can hydrate to form dihydrate gypsum crystals, making it a potential cement substitute [27].

Yang et al. [28] investigated the preparation of vegetation concrete by incorporating phosphogypsum (PG) into cement at different ratios (0%, 20%, 40%, and 60%). The results indicated that when the PG content was 20%, the compressive strength could be maintained above 10 MPa, while the pH value decreased to 9.5, achieving the optimal performance. Li et al. [29] found that adding PG to vegetation concrete could significantly promote the growth of Tall fescue. Moreover, when the PG content exceeded 20%, no additional alkalization treatment was required, as the pH value had already decreased to 8.5. Liu et al. [30] prepared vegetation concrete by mixing PG with electrolytic manganese residue, and the 14-day compressive strength and porosity reached 3.49 MPa and 24.5%, respectively. Gong et al. [31] added HPG to cement at various dosages and found that when the HPG content was 10%, the 28-day compressive strength of the composite paste could reach 49.8 MPa. Singh et al. [32] demonstrated that the appropriate addition of PG to soil could provide essential nutrients such as calcium and sulfur for crops, thereby improving the yield and quality of agricultural products. The aforementioned studies illustrate that PG can replace part of the cementitious material and is more beneficial to plant growth. However, the higher the PG dosage, the poorer the mechanical properties of the concrete.

Recycled aggregates (RAs) are derived from waste concrete, formed after crushing, screening, and impurity removal. They are one of the important directions for the utilization of construction solid waste. With the acceleration of urban renewal and transformation, the demolition of existing urban buildings will inevitably generate a large amount of construction waste: approximately 8000–13,000 tons of construction waste is produced per 10,000 m^2^ of building demolition [33], posing an arduous task for the resource utilization of construction solid waste.

Due to differences in raw material sources and compositions, the performance of recycled aggregates is discrete [34]. Moreover, the old mortar attached to the surface of recycled aggregates and the damage caused by mechanical processing increase their porosity and bulk void ratio, resulting in worse mechanical properties and durability compared to natural crushed stone (recycled aggregates have a higher crushing value, water absorption, and porosity, and lower apparent density) [35,36]. Therefore, concrete incorporating RA is inferior to OPC concrete in terms of workability [37], mechanical properties [38], and durability [39], which are the main factors limiting the popularization and application of RA.

Therefore, the natural acidity, cementitiousness, and fertilizer characteristics of hemi-hydrate phosphogypsum, as well as the light weight, porosity, and surface roughness of recycled aggregates, highlight the strong compatibility of these two materials in vegetation concrete. In this study, replacing part of ordinary Portland cement with hemi-hydrate phosphogypsum as a cementitious component not only meets the required mechanical properties compared with conventional cement-based vegetation concrete, but also achieves the effect of direct alkalinity reduction; furthermore, the incorporation of recycled aggregate realizes the resource reuse of these two solid waste materials. However, the weak cementitious effect of hemi-hydrate phosphogypsum, the weak interface of the residual mortar layer in recycled aggregates, and the inherent alkalinity of recycled aggregates directly affect the comprehensive properties of vegetation concrete.

Based on the above factors, this study used recycled aggregates to fully replace natural crushed stone, and HPG to replace cement in a gradient manner. The setting time, strength, porosity, pH value, and other properties of vegetation concrete were analyzed. Microscopic detection methods (SEM and XRD) were used to explore the internal hydration mechanism and optimize the mix ratio. Planting tests were conducted to evaluate the performance, providing technical support and a theoretical basis for the application of phosphogypsum–recycled aggregate vegetation concrete in engineering.

## 2. Materials and Methods

### 2.1. Raw Materials

#### 2.1.1. Cementitious Materials

**Figure 1:**
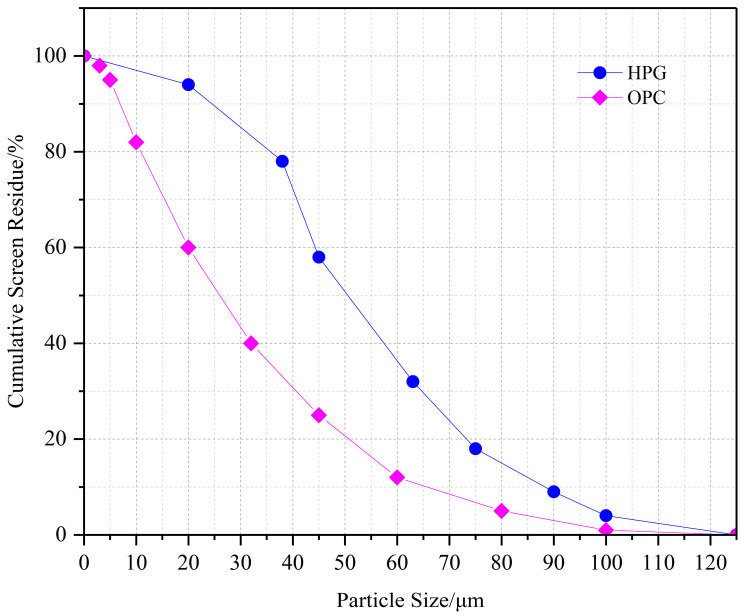
Particle size distribution Curves of OPC and HPG.

Cement (OPC): P.O 42.5 ordinary Portland cement was used. Its composition and characteristics are shown in Table 1, and the particle size distribution curve is shown in Figure 1.

Semi-hydrated phosphogypsum (HPG): Provided by a phosphating company in Guizhou. Waste phosphogypsum was screened (particle size < 0.15 mm) and drying-dehydrated with hot air at approximately 160 °C to form HPG, with the main component of CaSO_4_·0.5H_2_O. Its composition and characteristics are shown in Table 2, and the particle size distribution curve is shown in Figure 1.

#### 2.1.2. Coarse Aggregates

**Figure 2:**
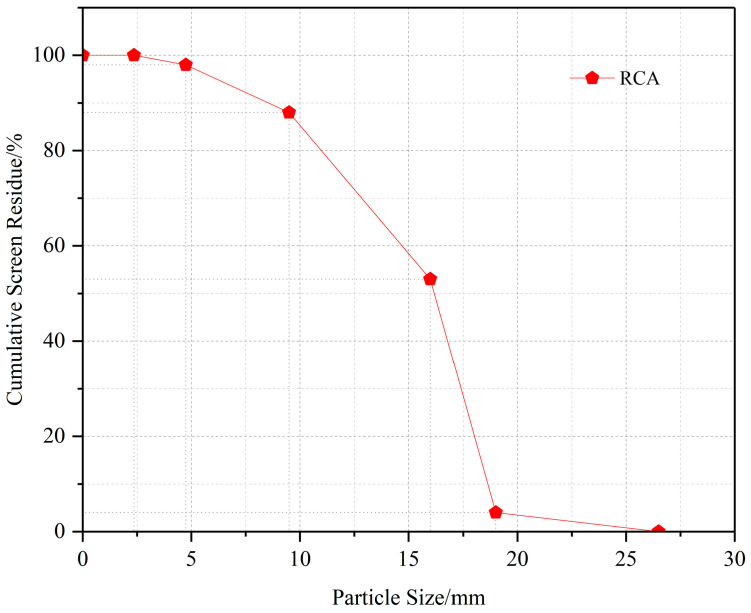
Recycled coarse aggregate gradation curves.

Recycled coarse aggregates (RCAs): Obtained from the concrete (C30) of a demolished old building floor slab. After mechanical crushing and screening, single-grade coarse aggregates with a particle size of 10–20 mm were prepared. The main physical properties are shown in Table 3, and the particle size distribution curve is shown in Figure 2.

#### 2.1.3. Admixtures

Silica fume (SF): Industrial grade, with SiO_2_ content > 96%, average particle size of 0.1–0.3 μm, and specific surface area of 22,000 m^2^/kg.

Sodium gluconate (SG): Analytical grade, with purity > 98%. Used as a retarder to solve the problem that HPG sets quickly when exposed to water, which affects the working performance of concrete [40].

Polycarboxylate superplasticizer (PCE): Solid content of 40%, with a water reduction rate of 25% when the admixture dosage is 1%. Used to alleviate concrete strength loss caused by high water absorption of HPG and coarse aggregates [41].

### 2.2. Mix Ratio Design

#### 2.2.1. Theoretical Method

The mix ratio of vegetation concrete is designed based on its structural performance, vegetation performance, and working performance. Common methods include the specific surface area method [42], volume method [43], and empirical test method [44]. In this study, the volume method was used to control the porosity parameter. The target value was the designed porosity; the RCA dosage was determined by the coarse aggregate packing theory [45], and the cementitious paste dosage was calculated based on the sum of the designed pore volume and coarse aggregate volume, as shown in Equations (1)–(3):

(1) $${W_{G}\;=\;\rho }_{G,c}\;\times \;\alpha$$

W_G_: Dosage of coarse aggregate per unit volume (kg/m^3^);
ρ_G,c_: Compacted bulk density of recycled coarse aggregates (kg/m^3^);
α: Reduction coefficient, taken as 0.98.

(2) $$W_{J}=(1\;-\;\frac{W_{G}}{\rho_{G}}\;-\;R_{\mathrm{viod}})\;\times \;\rho_{l}$$

W_J_: Dosage of cementitious paste per unit volume of concrete (kg/m^3^);
ρ_G_: Apparent density of coarse aggregates (kg/m^3^);
R_void_: Designed porosity value (decimal);
ρ_l_: Density of cementitious paste (kg/m^3^).

(3) $$W_{B}=\frac{W_{J}}{1+W/B}$$

W_B_: Dosage of cementitious materials per unit volume of concrete (kg/m^3^);
W/B: Water–binder ratio (decimal).

#### 2.2.2. Test Mix Ratio

The vegetation concrete mix ratio was designed with a porosity of 24%, and recycled aggregates fully replaced natural aggregates.

Group A: HPG replaced cement at mass ratios of 0%, 25%, 50%, and 75% to study the effect of HPG on the performance of vegetation concrete.

Group B: Sodium gluconate was added at mass ratios of 0%, 0.1%, 0.3%, 0.5%, and 0.8% to study the effect of sodium gluconate retarder on the setting time of HPG (at a replacement rate of 50%).

The mix ratio of vegetation concrete is shown in Table 4.

### 2.3. Test Methods

#### 2.3.1. Specimen Preparation

**Figure 3:**
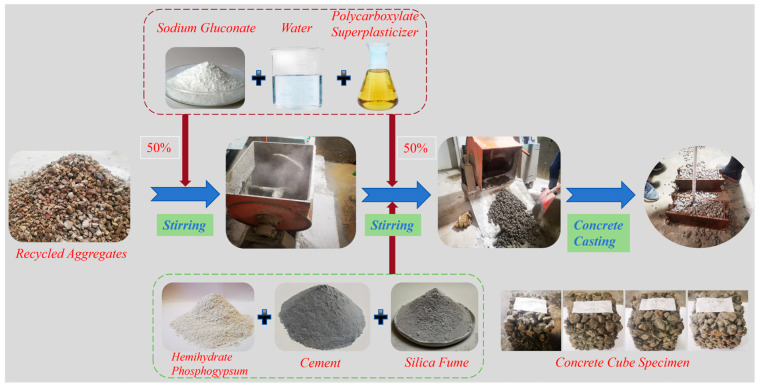
Preparation of vegetated concrete test blocks.

Concrete was prepared using the stone-coating method [46]: all recycled coarse aggregates, 50% of water, and admixtures were first added and mixed, followed by the gradual addition of pre-mixed cementitious materials (HPG, OPC, SF) and the remaining water and admixtures. This mixing process facilitates the full and uniform coating of aggregates with paste, improving the overall strength (Figure 3).

To avoid excessive water absorption by the recycled coarse aggregates during mixing, a pre-saturated water absorption treatment was adopted [47], which is beneficial for improving the later-stage strength of concrete. A forced single-shaft horizontal mixer was used for mixing. The mixed concrete was poured into 100 mm × 100 mm × 100 mm molds and then manually rodded [48], followed by light compaction using a wood float; this operation helps mitigate slurry sedimentation and ensures the formation of interconnected pores.

#### 2.3.2. Mechanical Performance and Setting Time Test

**Compressive strength test:** Conducted in accordance with the GB/T 50081-2019 [49], using a concrete pressure testing machine (Model YAW-3000, Jinan Shijin Group Co., Ltd., Jinan, Shandong Province, China). Three parallel cube specimens (100 mm × 100 mm × 100 mm) were used per group, and the test was repeated three times.

**Setting time test:** Measured with a Vicat apparatus (Model WX-2000, Tianjin Jianzhu Instrument & Testing Machine Co., Ltd., Tianjin, China) in accordance with the GB/T 1346-2011 [50]. Each group included three parallel samples, and the test was repeated three times.

#### 2.3.3. Pore pH Value Test

Common methods for testing the pore pH value of vegetation concrete include the solid–liquid extraction method, alkalinity release method, and acid–base neutralization method. The pH value measured by the solid–liquid extraction method is higher than the actual pH value in concrete pores [51]. Wang et al. [52] pointed out that the result obtained by the alkalinity release method is closer to the actual pore pH value of concrete.

In this study, the alkalinity release method was used: a cubic concrete block with a side length of 100 mm was placed in a container filled with 4000 mL of pure water. After soaking for 4 h, the water sample was replaced until the pH value stabilized [53]. The pH value of the aqueous solution was regarded as the pore pH value of concrete, measured using an electronic pH meter (Model DLX-PH-05-1, Delixi Electric Co., Ltd., Liushi Town, Yueqing City, Zhejiang Province, China). Three parallel concrete cubic samples were prepared per experimental group, and the entire soaking and pH measurement process was repeated three times.

#### 2.3.4. Porosity Test

Porosity was determined by the drainage method: 28-day-old vegetation concrete specimens were immersed in water for 2 h. After full water absorption, the specimens were taken out, the surface water was drained, and they were placed in a measuring cylinder filled with water. The volume of drained water was measured, and the porosity P was calculated using Equation (4):

(4) $$P=(\frac{L_{0}\;-\;L_{1}}{L_{0}})\;\times \;100\%$$

L_0_: Volume of the concrete cube specimen (mm^3^; the specimen used in this study was a cube with a side length of 100 mm);
L_1_: Volume of drained water (mm^3^).

Three parallel 28-day-old specimens were used per group, with one drainage test performed per specimen.

#### 2.3.5. Microscopic Test

To analyze the hydration process of HPG–recycled aggregate-based vegetation concrete in detail, SEM and XRD were used to characterize the micromorphology and mineral phases of the specimens. The test samples were the cementitious materials of Group A at different ages (3d–28d). Due to the concentrated testing schedule, after sampling, the samples were soaked in anhydrous ethanol for 48 h and then dried at 60 °C to eliminate test errors caused by the self-hydration of materials.

XRD test: Conducted using an Advance D8 diffractometer (Model Advance D8, Bruker AXS GmbH, Karlsruhe, Baden-Württemberg, Germany); samples were ground into powder with a particle size of ≤200 mesh (<75 μm).

SEM test: Conducted using a Zeiss EVO 15 microscope (Model EVO 15, Carl Zeiss AG, Oberkochen, Baden-Württemberg, Germany); samples were cut into 5 mm × 5 mm × 5 mm blocks.

## 3. Results and Discussion

### 3.1. Setting Time of Vegetation Concrete

HPG has an extremely fast setting rate: during hydration, it generates dihydrate calcium sulfate crystals (CaSO_4_·2H_2_O), which grow and overlap rapidly to form a structure. A high content of H^+^ easily causes the “flash set” of HPG [54], which cannot meet the working time required for construction. Sodium gluconate can inhibit the hydration process by adsorbing on the particle surface [55], so it was used as a retarder to control the setting time in this study.

#### 3.1.1. Effect of HPG Replacement Rate on Setting Time (Group A)

**Figure 4:**
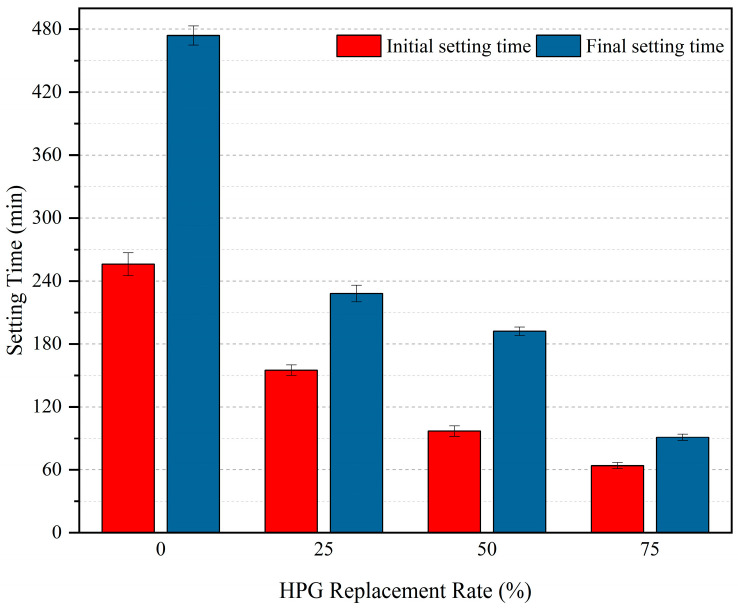
Effect of HPG content on setting time of cementitious materials.

Figure 4 shows the initial and final setting times of concrete with different HPG replacement rates (with a fixed sodium gluconate dosage of 0.5%). With the increase in HPG replacement rate, both the initial and final setting times of the vegetation concrete are significantly shortened: the initial setting time decreases from 256 min (0% HPG) to 64 min (75% HPG), and the final setting time reduces from 474 min to 91 min. Specifically, at a replacement rate of 25%, the initial setting time is 155 min (39.4% shorter than that of the reference group HPG = 0%) and the final setting time is 228 min (51.9% shorter than that of HPG = 0%); at a replacement rate of 75%, the initial setting time is 64 min (75% shorter than that of HPG = 0%) and the final setting time is 91 min (80.8% shorter than that of HPG = 0%). One-way analysis of variance (ANOVA) indicates that there are extremely significant differences in the initial setting time (F = 18.75, *p* < 0.01) and final setting time (F = 23.42, *p* < 0.01) among groups with different replacement rates. This confirms that the HPG content has a statistically significant effect on the setting time of the concrete, and the setting acceleration effect is more pronounced at high replacement rates.

These results indicate that under the condition of a fixed retarder dosage, the HPG content determines the setting time of the composite cementitious material: the higher the HPG dosage, the shorter the setting time. In addition, the hydration of HPG takes precedence over that of cement and dominates the initial reaction stage. Replacing cement with HPG accelerates the hydration rate of the composite cementitious material, which can provide support for the early strength of concrete.

#### 3.1.2. Effect of Sodium Gluconate Dosage on Setting Time (Group B)

**Figure 5:**
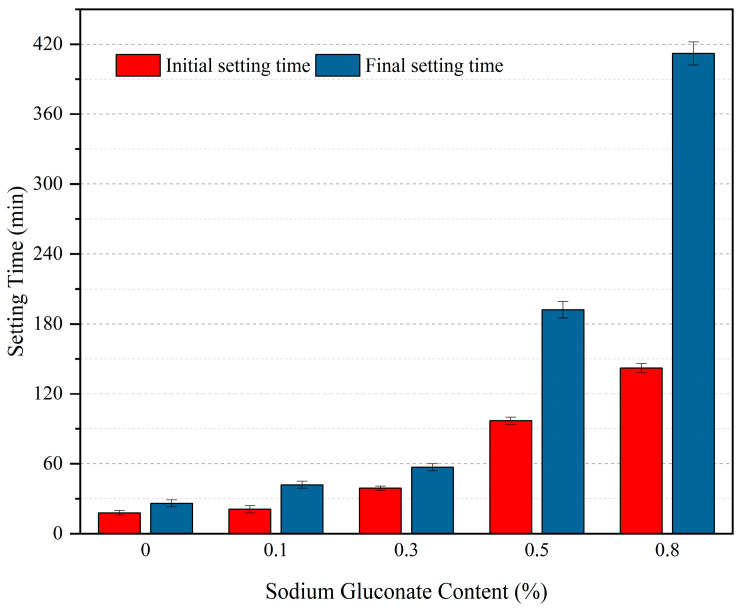
Effect of sodium gluconate content on setting time of cementitious materials.

Figure 5 shows the initial and final setting times of concrete with different SG dosages (with a fixed HPG replacement rate of 50%). With increasing SG dosage, both initial and final setting times showed a linear increasing trend. Without SG: Initial setting time = 18 min, final setting time = 26 min (fast setting, reflecting the hydration characteristics of HPG, but the “flash set” of pure HPG was alleviated). At an SG dosage of 0.5%: Initial setting time = 97 min, final setting time = 192 min (meeting the construction working time requirement). At an SG dosage of 0.8%: Setting time was further prolonged. Statistical analysis via one-way ANOVA demonstrates extremely significant variations in initial setting time (F = 27.86, *p* < 0.01) and final setting time (F = 31.54, *p* < 0.01) across different SG content groups. This validates the statistically significant regulatory role of SG content in concrete setting time, where the retarding effect becomes more prominent at elevated dosages.

These results indicate that SG can effectively regulate the setting time of the composite cementitious material (OPC + HPG). When the dosage is >0.5%, the initial setting time exceeds 1 h, and the final setting time is within 5 h, which can meet the construction requirements well.

### 3.2. Compressive Strength of Vegetation Concrete

The vegetation concrete specimens in Group A (with different HPG replacement rates) exhibited good dry hardness. The aggregates and cementitious materials formed a stable skeleton structure without pore blockage caused by the flow of cementitious materials.

#### 3.2.1. Effect of HPG Replacement Rate on Compressive Strength

**Figure 6:**
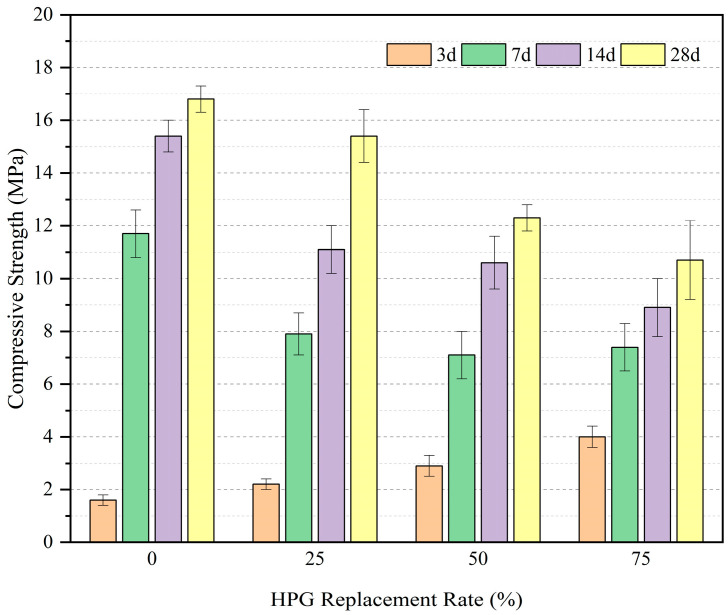
Development of compressive strength of concrete with age.

Figure 6 shows the effect of the HPG replacement rate on compressive strength. The effects of the increase in HPG replacement rate were as follows:

Early strength (3 d): Significantly increased. The compressive strength of PVC1 (0% HPG) was only 1.6 MPa, while that of PVC2 (25% HPG), PVC3 (50% HPG), and PVC4 (75% HPG) increased to 2.2 MPa, 2.9 MPa, and 4 MPa, respectively. This indicates that a higher HPG dosage contributes more to the early strength of concrete.

Mid–late strength (7 d–28 d): The growth rate gradually decreased. After 3d, the strength growth rate of PVC1 accelerated: its 7 d strength exceeded that of other groups, and its 28 d strength reached 16.8 MPa. In contrast, the 28 d strengths of PVC2, PVC3, and PVC4 decreased to 15.4 MPa, 12.3 MPa, and 10.7 MPa, respectively.

Overall, with increases in HPG replacement ratio, the compressive strength of vegetation concrete at all ages first increases and then decreases. One-way ANOVA indicates that there are significant differences in the compressive strength at 3 d (F = 12.36, *p* < 0.05), 7 d (F = 8.72, *p* < 0.05), 14 d (F = 10.58, *p* < 0.05), and 28 d (F = 15.63, *p* < 0.01) among groups with different replacement ratios. Among these, the differences in 28 d compressive strength between the 0% replacement group (PVC1) and the 50% (PVC3) and 75% (PVC4) replacement groups reach an extremely significant level, which also illustrates that HPG content has a considerable impact on the late-stage strength of vegetation concrete. This phenomenon is attributed to the accelerated hydration of cement in the mid–late stage, which promotes strength development. However, an increase in the HPG replacement ratio reduces the cement dosage, thereby limiting the improvement of late-stage strength. This phenomenon is attributed to the accelerated hydration of cement in the mid–late stage, which promotes strength growth. However, an increase in HPG replacement rate reduces the cement dosage, limiting the later-stage strength improvement.

#### 3.2.2. Fracture Surface Analysis of Vegetation Concrete

**Figure 7:**
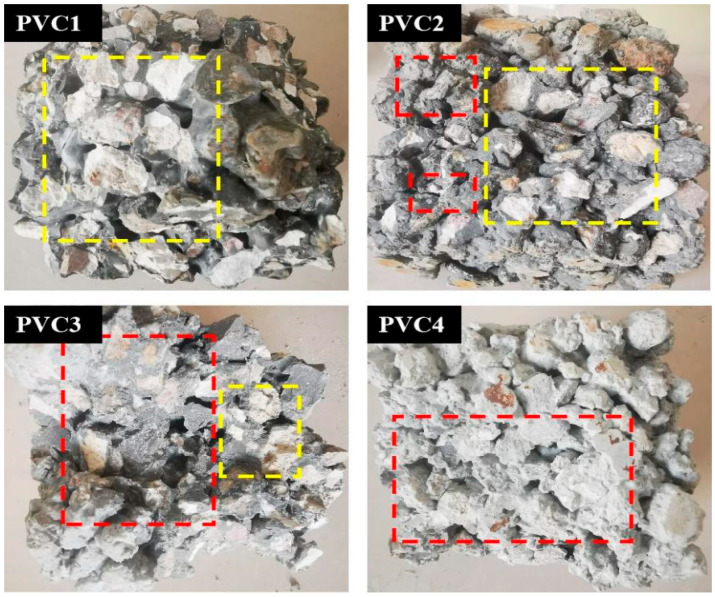
Fracture surfaces of vegetated concrete specimens.

Figure 7 shows the fracture surfaces of vegetation concrete specimens:

PVC1 (0% HPG): Most fractures were the splitting of recycled aggregates, indicating that the coating strength of the paste was higher than the strength of recycled aggregates.

PVC2 (25% HPG): More interface damage between recycled aggregates and paste in the transition zone occurred, with a small amount of recycled aggregate fracture.

PVC3 (50% HPG) and PVC4 (75% HPG): Fractures were mainly interface damage between recycled aggregates and paste in the transition zone.

These results suggest that with a low HPG dosage, the paste has a more obvious coating effect, and recycled aggregates have a greater impact on concrete strength. With the increase in HPG dosage, the cementitious coating effect weakens, and the impact of recycled aggregates on strength gradually decreases. Therefore, when preparing vegetation concrete with a high HPG dosage, the difference in the effect of natural aggregates and recycled aggregates on strength is smaller, and recycled aggregates are more cost-effective.

#### 3.2.3. Effect of Sodium Gluconate on Compressive Strength

**Figure 8:**
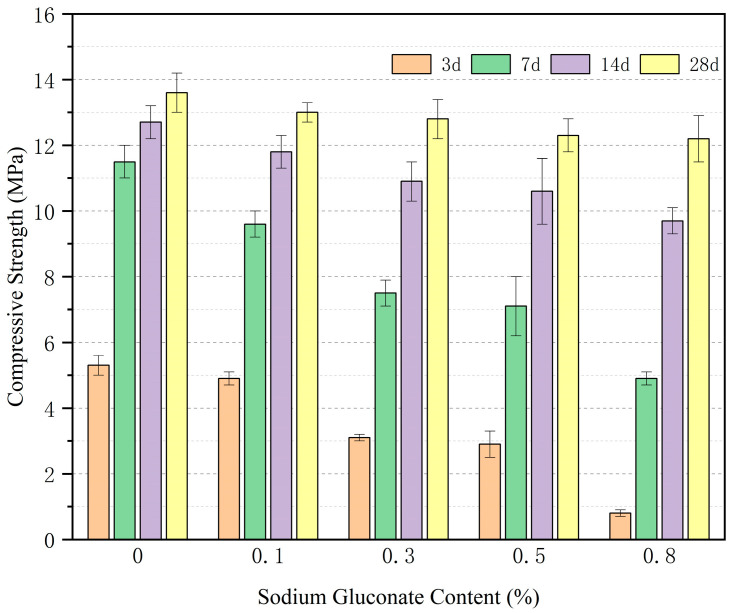
Effects of sodium gluconate content on strength.

Figure 8 shows the effect of sodium gluconate dosage on compressive strength (with a fixed HPG replacement rate of 50%). With the increase in sodium gluconate dosage from 0 to 0.8%, the compressive strength of vegetation concrete at all ages shows an overall decreasing trend, with the early-age strength (3 d) being more significantly affected: the 3 d compressive strength decreases from 5.3 MPa (when SG = 0%) to 0.8 MPa (when SG = 0.8%), representing a reduction of 84.9%; the 28 d compressive strength gently decreases from 13.6 MPa to 12.2 MPa, with only a 10.3% reduction. One-way ANOVA indicates that there are significant differences in the compressive strength at 3 d (F = 22.35, *p* < 0.01), 7 d (F = 15.82, *p* < 0.01), 14 d (F = 9.67, *p* < 0.05), and 28 d (F = 6.78, *p* < 0.05) among groups with different SG dosages. This confirms that the effect of SG dosage on the compressive strength of concrete is statistically significant, and the inhibitory effect on early-age strength is more pronounced. Therefore, a reasonable sodium gluconate dosage can meet the requirements of setting time and early strength.

### 3.3. Alkalinity of Vegetation Concrete

**Figure 9:**
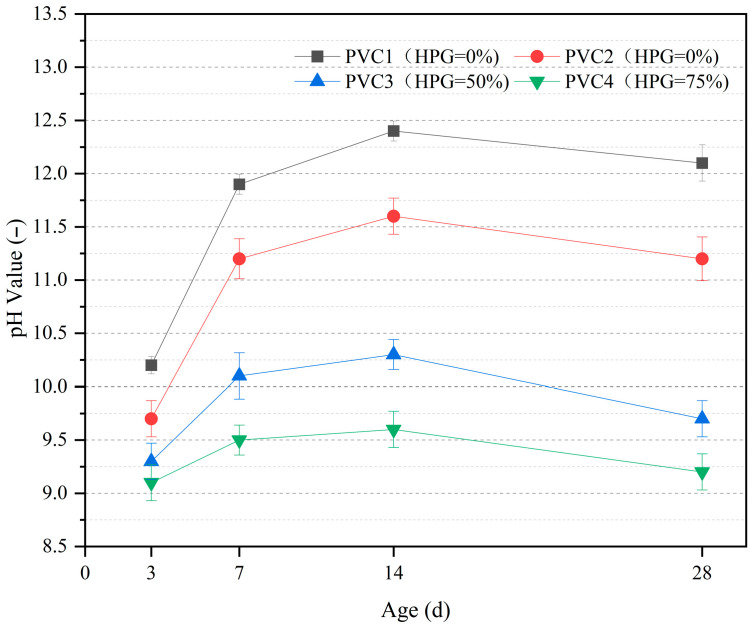
Effects of HPG content on pore pH of vegetated concrete.

Figure 9 shows the effect of HPG dosage on the leachate pH value of vegetation concrete. With increases in HPG dosage, the pH value significantly decreased. At 3 d of age, the pH value decreased from 10.2 to 9.1 (a decrease of 10.8%); At 28 d of age, the pH value decreased from 12.1 to 9.2 (a decrease of 24%). This confirms that HPG has a good alkali-regulating ability: the higher the HPG dosage, the more significant the alkali-reducing effect. The reason for the alkali reduction is mainly that HPG itself is weakly acidic, which can quickly neutralize the OH^−^ released by cement hydration products, reducing the pH of the pore solution. At the same time, the increase in HPG replacement rate means a decrease in cement dosage, resulting in less OH^−^ released by hydration products, thus reducing the pH value.

The pH development with age shows that the pH change in each group presents a “first increase and then decrease” trend with age:

- Early stage (3 d): Low pH value. HPG hydration dominates, while cement hydration is inhibited (resulting in less OH^−^ generation). Additionally, a small amount of acidic substances in HPG react with Ca(OH)_2_ (a cement hydration product), consuming part of the OH^−^.
- Mid-stage (14 d): pH value reaches the peak. Cement hydration is enhanced, and the generation of Ca(OH)_2_ exceeds its consumption, leading to the accumulation of OH^−^.
- Late stage (28 d): pH value slightly decreases and stabilizes. This may be due to weakened cement hydration or the secondary hydration reaction between the silica fume (an alkali-active material) and Ca(OH)_2_ (resulting in higher OH^−^ consumption than generation).

Plants generally prefer a near-neutral environment. Excessively high alkalinity (e.g., pH = 12.1 for 0% HPG) may inhibit root development and microbial activity. However, excessively low pH value (e.g., pH = 9.2 for 75% HPG) will cause cement hydration products (e.g., C-S-H) to become unstable, precipitate, and crystallize, reducing the system strength [56]. Therefore, maintaining the pH value of vegetation concrete at approximately 10 is reasonable. The results show that increasing the HPG replacement rate can effectively reduce the alkalinity of concrete, making the 28-day pH value closer to the neutral range, which is beneficial for creating a suitable vegetation environment. Additionally, the pH value gradually decreases with age and stabilizes, providing a relatively stable chemical environment for later plant colonization. However, the HPG replacement rate must be controlled to balance strength performance.

### 3.4. Porosity of Vegetation Concrete

**Figure 10:**
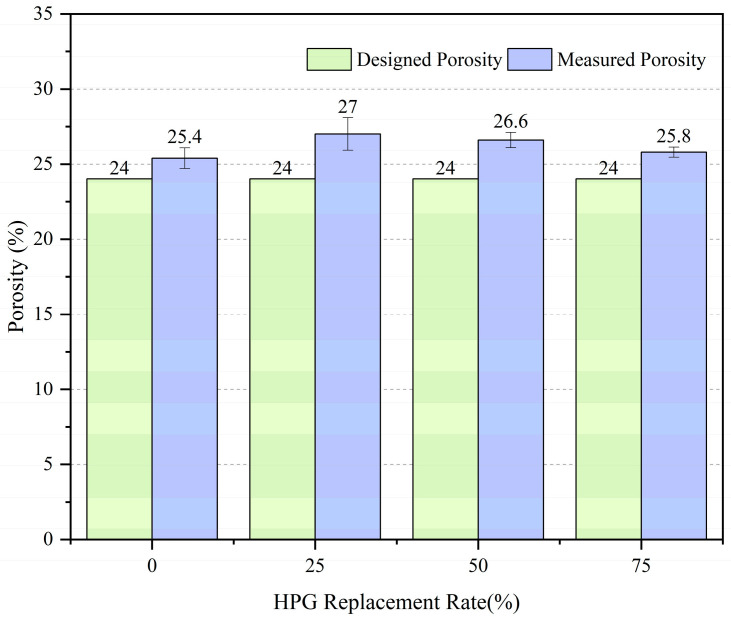
Porosity of vegetated concrete.

The designed porosity of vegetation concrete was 24%. Figure 10 shows the measured 28 d porosity of concrete (with fixed admixtures: 5% silica fume, 0.5% sodium gluconate, and 1% polycarboxylate superplasticizer). The porosity under different HPG replacement rates was higher than the designed porosity (24%), indicating that the vegetation concrete prepared by this mix system has sufficient pores.

Specifically, with the increase in HPG replacement rate from 0 to 25%, the porosity increased from 25.4% to 27.0%. This is because the interaction between HPG, cement, and silica fume is more conducive to pore formation at a 25% replacement rate. When the replacement rate continues to increase to 50% and 75%, the porosity gradually decreased to 26.6% (50% replacement rate) and 25.8% (75% replacement rate), but remained higher than that of the 0% replacement group. This may be due to the adjustment of the paste hydration process or particle packing state by a high HPG replacement rate, which weakens the promotion effect on pore formation. A high porosity is beneficial for the vegetation functions of vegetation concrete (e.g., air permeability and water retention).

### 3.5. Analysis of Hydration Products

**Figure 11:**
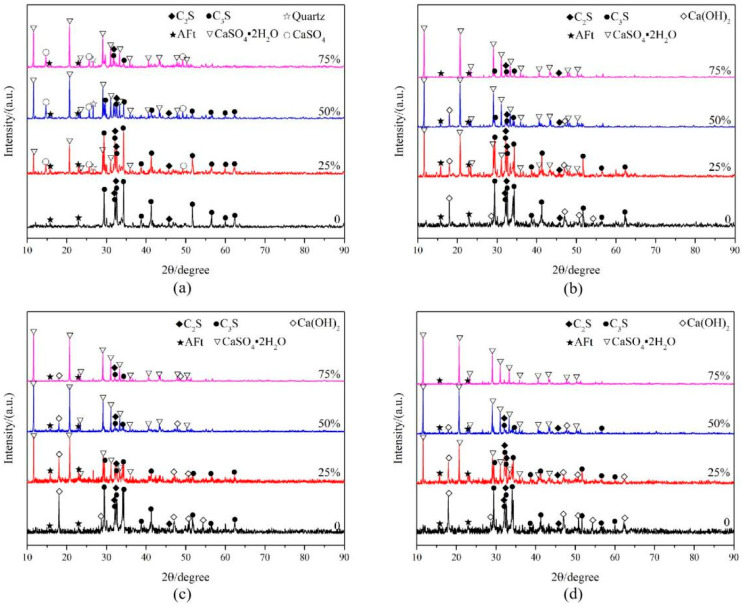
Comparison of hydration products of hemi-hydrate phosphogypsum with different replacement rates. (**a**) Age 3 d; (**b**) age 7 d; (**c**) age 14 d; (**d**) age 28 d.

Figure 11 shows the XRD patterns of cementitious materials prepared with different HPG replacement rates at various ages. The main hydration products were CaSO_4_·2H_2_O, AFt (ettringite), and Ca(OH)_2_, while CaSO_4_, Quartz, C_2_S, and C_3_S were raw material components.

#### 3.5.1. Hydration Products at Early Age (3 d)

Figure 11a shows that at 3 d age, the peak intensity of CaSO_4_·2H_2_O increased with the increase in HPG content. CaSO_4_·2H_2_O is the hydration product of HPG (Equation (5)), indicating that HPG was extensively hydrated in the early stage.

(5) $$CaSO_{4}\cdot 0.5H_{2}O+1.5H_{2}O\to CaSO_{4}\cdot 2H_{2}O$$

Additionally, AFt was detected in all groups. AFt is the product of the reaction between CaSO_4_ and C_3_A (in cement) (Equation (6)). The initial AFt peak intensity was low and changed slightly with age, indicating that CaSO_4_·2H_2_O (generated by HPG hydration) did not promote AFt formation.

(6) $$3CaO\cdot Al_{2}O_{3}+3CaSO_{4}\cdot 2H_{2}O+26H_{2}O\to 3CaO\cdot Al_{2}O_{3}\cdot 3CaSO_{4}\cdot 32H_{2}O$$

**Figure 12:**
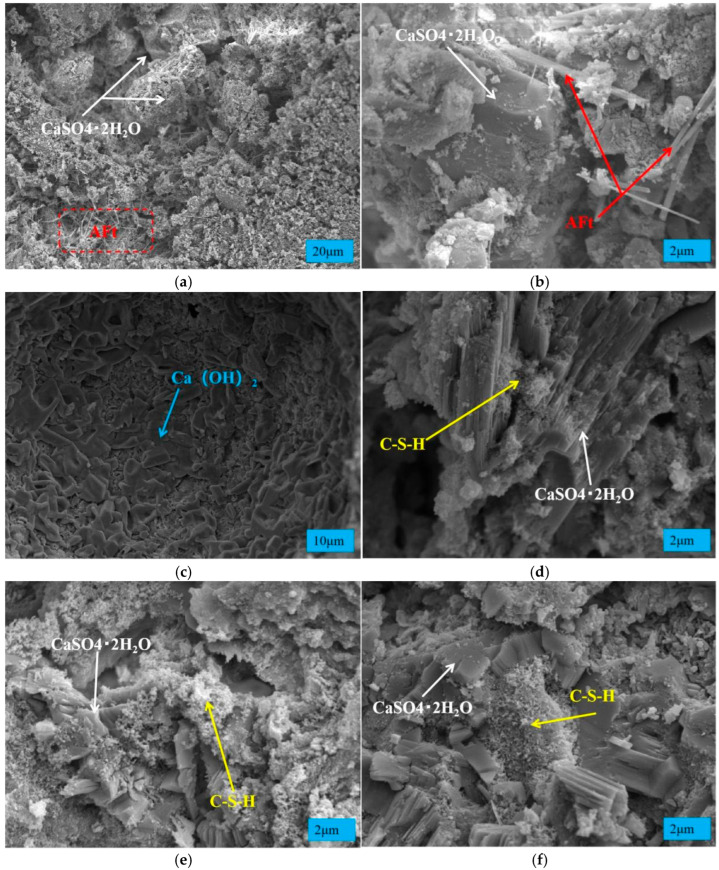
SEM morphology of HPG at a replacement rate of 50%. (**a**,**b**) Age 3 d; (**c**,**d**) age 7 d; (**e**) age 14 d; (**f**) age 28 d.

SEM images (Figure 12a,b) show that CaSO_4_·2H_2_O mainly exists as plate-like crystals, coexisting with acicular AFt crystals. These crystals form a framework structure in the early stage of concrete, which is the main source of early strength. This confirms the strength test results: the early strength of concrete with HPG is higher than that without HPG, and increases with the HPG content.

#### 3.5.2. Hydration Products at Mid–Late Age (7 d–28 d)

In the XRD patterns, the diffraction peaks at 18.0°, 47.2°, and 50.9° correspond to Ca(OH)_2_ (CH). CH is one of the hydration products of C_2_S and C_3_S (in cement) (Equations (7) and (8)):

(7) $$2(2CaO\cdot SiO_{2})+4H_{2}O\to 3CaO\cdot 2SiO_{2}\cdot 3H_{2}O+Ca{\left(OH\right)}_{2}$$

(8) $$3CaO\cdot SiO_{2}+6H_{2}O\to 3CaO\cdot 2SiO_{2}\cdot 3H_{2}O+3Ca{\left(OH\right)}_{2}$$

Figure 11b,c show that CH was not detected in each group at 3 d age or its content was very small, but CH began to appear at 7 d, and the peak value was the strongest at 14 d, and decreased with the increase in HPG content. This indicates that cement hydration is inhibited in the early stage (3 d) and progresses slowly—even in the 0% HPG group. This phenomenon is attributed to sodium gluconate (a retarder), which effectively controls the setting time of the cementitious material but also inhibits the early hydration of cement. Cement hydration is enhanced in the mid-stage (7 d–14 d).

Figure 11d shows that at 28 d age, the CH peak intensity weakened and decreased with the increase in HPG content, and the CH peak disappeared when the HPG replacement rate was 75%. The reason may be that the cement hydration rate slows down or the raw material silica fume (SiO_2_) consumes CH (Equation (9)). At the same time, the decrease in cement content is also the main reason for the decrease in CH generation. The content of CH directly affects the pore pH value of vegetation concrete, and its development trend is consistent with the pH value change law of vegetation concrete, which also confirms the previous conclusion.

(9) $$Ca{\left(OH\right)}_{2}+SiO_{2}+H_{2}O\to CaO\cdot SiO_{2}\cdot H_{2}O$$

#### 3.5.3. C-S-H (Main Source of Later-Stage Strength)

The generation of CH indicates the formation of C-S-H (calcium silicate hydrate), the main hydration product of cement and the key source of cement strength. SEM images (Figure 12c,d) show that CH exists as flaky crystals, while C-S-H exists as a flocculent gel. At 3 d of age, only a small amount of C-S-H is generated, and the accompanying CH may react with the acidic “H^+^” in semi-hydrated phosphogypsum to consume CH. Until the middle stage (7 d–14 d), a large amount of flocculent C-S-H gel is generated, gathering between the CaSO_4_·2H_2_O crystals (Figure 12e). When the HPG replacement rate is 50%, a large amount of C-S-H is interleaved in the CaSO_4_ crystals at 28 d of age (Figure 12f), playing a filling and compacting role, providing support for the later strength development of concrete, which also confirms the later strength development law of vegetation concrete: the concrete without HPG has a faster later strength growth and higher strength.

### 3.6. Growth Performance of Plants

**Figure 13:**
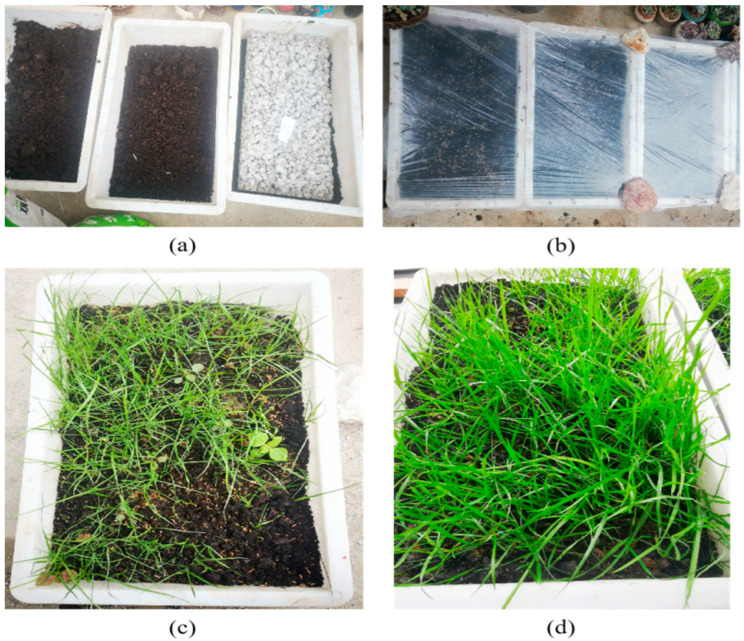
Planting test. (**a**) Vegetated concrete board; (**b**) soil covering and seeding; (**c**) 30 d growth status; (**d**) 60 d growth status.

The mix ratio of Group A with a 50% HPG replacement rate (designed porosity = 24%) was selected for the planting test. Vegetation concrete slabs (50 mm × 150 mm × 250 mm) were prepared, and 3 cm thick organic fertilizer soil layers were paved on the bottom and surface of the slabs. Tall fescue seeds were sown, and the test was conducted outdoors at 15–28 °C in early September. The test results are shown in Figure 13.

**Figure 14:**
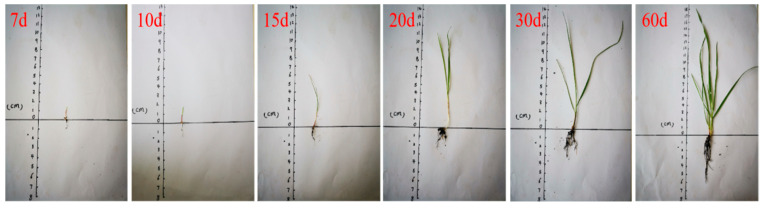
Vegetation growth detection.

**Figure 15:**
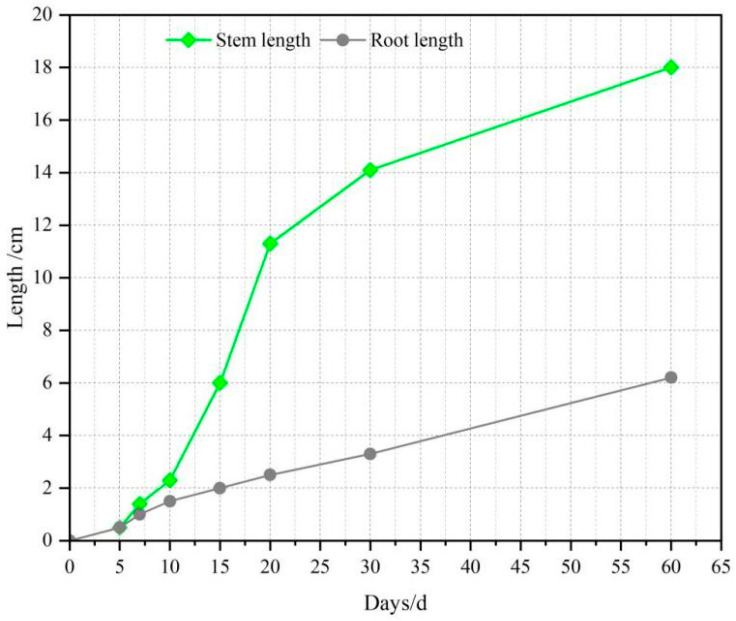
Plant growth records.

**Figure 16:**
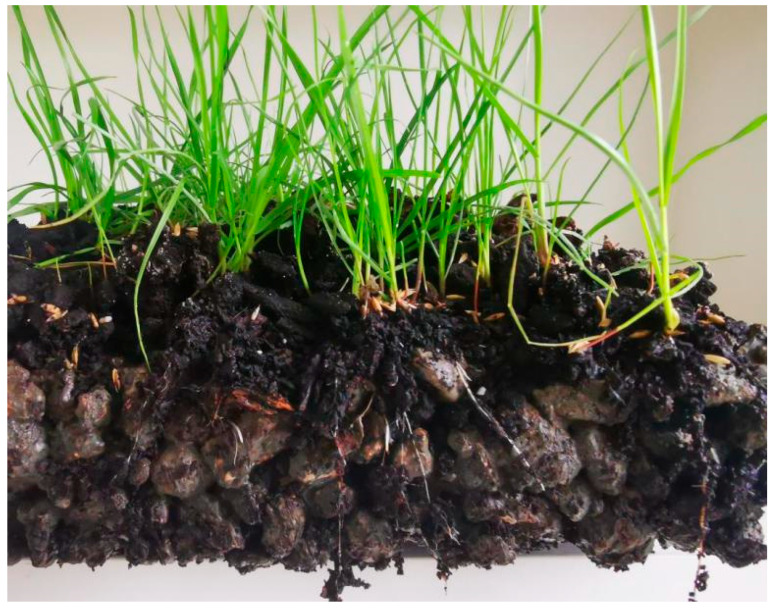
Growth status of plant root system (60 d).

Growth Status of Tall Fescue:

- Germination and lawn formation: Tall fescue germinated at approximately 5 d and formed a lawn at 30 d, with a coverage rate of >85% (Figure 13).
- Plant height: 2–3 cm at 10 d, 15 cm at 30 d, and 18 cm at 60 d. No lodging or yellowing occurred (Figure 14).
- Root growth: 2–3 cm at 20 d (with increased fibrous roots), 4 cm at 30 d (well-developed fibrous roots), and 6–8 cm at 60 d. Some roots grew along the sidewalls of concrete pores and penetrated the 5 cm thick vegetation concrete slab, extending into the underlying soil (Figure 15 and Figure 16).

These results indicate that the vegetation concrete with a 50% HPG replacement rate and 24% porosity provides a good growth environment for Tall fescue.

## 4. Conclusions

To realize the resource utilization of HPG solid waste and address the technical bottlenecks of traditional vegetation concrete (e.g., excessively high alkalinity and poor adaptability of recycled aggregates), this study systematically investigated the effects of the HPG replacement ratio (substituting cement) and sodium gluconate dosage on the mechanical properties, alkalinity environment, hydration mechanism, and plant growth adaptability of RCA-based vegetation concrete. The correlation laws between material composition and comprehensive performance were clarified, and the optimal mix proportion was determined. The main conclusions are as follows:

- For vegetation concrete using HPG to replace cement (as cementitious material) and recycled aggregates to replace natural crushed stone (as coarse aggregates): With increases in HPG replacement rate, the compressive strength gradually decreases, but the alkali-regulating effect is significant. When the porosity is 24% and the HPG content is 50%, the vegetation concrete exhibits optimal performance: the 28-day compressive strength reaches 12.3 MPa, and the pH value is 9.7 (19.8% lower than that of the 0% HPG group). Notably, compared with existing studies where the phosphogypsum content is typically 20%, this study increases the HPG content to 50% while maintaining a compressive strength exceeding 10 MPa and incorporating recycled aggregates. These results demonstrate that the proposed vegetation concrete possesses favorable mechanical properties and a suitable alkaline environment for plant growth.
- When recycled aggregates are used as coarse aggregates: With increases in HPG replacement rate, the impact of recycled aggregates on the strength of vegetation concrete decreases. When the HPG replacement rate <50%, concrete damage is mainly manifested as recycled aggregate fracture, and concrete strength can be improved by enhancing aggregate strength. When the HPG replacement rate >50%, concrete damage is mainly manifested as paste fracture, and recycled aggregates are more suitable than natural crushed stone.
- The addition of sodium gluconate can effectively control the “flash set” of HPG, but has a significant impact on the early hydration of cement. When the HPG content is 50%, adding 0.5% sodium gluconate as a retarder results in an initial setting time of 97 min and a final setting time of 192 min, which meets construction requirements with little influence on later-stage strength. Microscopic analysis shows that the early strength (3 d–7 d) of vegetation concrete is mainly contributed by CaSO_4_·2H_2_O crystals (HPG hydration product), while the later-stage strength is supplemented by C-S-H (cement hydration product). Additionally, C_2_S and C_3_S mineral phases exist in the system, which have potential for further hydration.
- Planting tests show that when the porosity is 24% and the HPG content is 50%, Tall fescue forms a lawn within 30 days; at 60 days, the plant height is 18 cm and the root length is 6–8 cm. Some roots grow along the sidewalls of concrete pores and penetrate the 5 cm thick vegetation concrete slab, with no lodging or yellowing, demonstrating good growth status.

## Figures and Tables

**Table 1:** Composition and characteristics of OPC.

Component (Mass %)						Setting Time (min)		Compressive Strength (MPa)
CaO	SiO_2_	Al_2_O_3_	Fe_2_O_3_	SO_3_	MgO	Initial Setting	Final Setting	
63.2	21.5	5.4	3.8	2.3	1.7	185	245	45.5

**Table 2:** Composition and characteristics of HPG.

Component (Mass %)							Setting Time (min)		Dry Strength (MPa)	pH
MgO	Na_2_O	P_2_O_5_	F	CaSO_4_·2H_2_O	CaSO_4_·0.5H_2_O	CaSO_4_	Initial Setting	Final Setting		
0.014	0.006	0.072	0.041	5.1	84.97	1.8	5	11	12.8	3.8

**Table 3:** Basic properties of RCA.

Gradation/mm	Apparent Density/(kg/m^3^)	Bulk Density/(kg/m^3^)	Water Absorption Rate/%	Crushing Value/%
10–20	2556	1327	4.1	15.6

**Table 4:** Mortar mix proportions.

Mix Group	No.	HPG Replacement Rate (%)	RCA(kg/m^3^)	HPG(kg/m^3^)	OPC(kg/m^3^)	SF(kg/m^3^)	SG (%)	PCE (%)	W/B
Group A	PVC1	0	1300	0	458	24	0.5	1	0.24
	PVC2	25	1300	115	343	24	0.5	1	0.27
	PVC3	50	1300	229	229	24	0.5	1	0.30
	PVC4	75	1300	343	115	24	0.5	1	0.33
Group B	PVC5	50	1300	229	229	24	0	1	0.30
	PVC6	50	1300	229	229	24	0.1	1	0.30
	PVC7	50	1300	229	229	24	0.3	1	0.30
	PVC8	50	1300	229	229	24	0.5	1	0.30
	PVC9	50	1300	229	229	24	0.8	1	0.30

## Data Availability

The original contributions presented in this study are included in the article. Further inquiries can be directed to the corresponding author.
